# Temporal heterogeneity of the root microbiome in *Panax ginseng* soils across ecological compartments under mild soil disturbance

**DOI:** 10.3389/fmicb.2024.1340575

**Published:** 2024-06-11

**Authors:** Zhenting Shi, Limin Yang, Meiling Yang, Kexin Li, Li Yang, Mei Han

**Affiliations:** Cultivation Base of State Key Laboratory for Ecological Restoration and Ecosystem Management, College of Traditional Chinese Medicine, Jilin Agricultural University, Changchun, China

**Keywords:** *Panax ginseng*, biodiversity, microhabitat spatial heterogeneity, taxonomic diversity, correlations, sustainable development

## Abstract

**Introduction:**

Knowledge on spatiotemporal heterogeneity of plant root microbiomes is lacking. The diversity of the root microbiome must be revealed for understanding plant–microbe interactions and the regulation of functionally crucial microbial taxa.

**Methods:**

We here investigated the dynamics of microbial group characteristics within each soil ecological compartment [rhizoplane (B), rhizosphere (J), and bulk soil (T)] across different cultivation years (year 4: F4 and year 5: F5) by using high-throughput sequencing (16S and ITS).

**Results:**

According to the species diversity, microbiome diversity and the ASV (amplified sequence variant) number in the rhizoplane ecotone increased significantly with an increase in the planting years. By contrast, the microbiome diversity of the rhizosphere soil remained relatively stable. PCoA and PERMANOVA analyses revealed that microbial taxa among different planting years and ecological compartments varied significantly. Planting years exerted the least effect on the rhizosphere microbiome, but their impact on fungi in the rhizoplane and bacteria in the bulk soil was the most significant.

**Discussion:**

Planting years influenced the microbial community composition in various ecological compartments of ginseng root soil. Potentially harmful fungi such as *Cryptococcus* (2.83%), *Neonectria* (0.89%), *llyonectria* (0.56%), *Gibberella* (0.41%), *Piloderma* (4.44%), and *Plectosphaerella* (3.88%) were enriched in F5B with an increase in planting years, whereas the abundance of potentially beneficial *Mortierella* increased. Correlation analysis indicated associations between bacterial taxa and soil pH/S-CAT, and between fungal taxa and soil moisture content/total potassium. Our study highlights the significance of changes in rhizoplane fungi and the stability of the rhizosphere microbial community in comprehending plant ecological sustainability.

## 1 Introduction

Various microorganisms, including bacteria and fungi, dwell in different soil ecological compartments in plant root systems. These microbes are pivotal players in plant growth, health, and productivity within cultivated ecological frameworks (Philippot et al., 2013; Lu et al., 2018; Li et al., 2019; Zhang G. Z. et al., 2022). Soil conditions and climate are some factors that collaboratively forge soil microbial communities, thereby serving as a microbial seed bank determining the microbial groups exposed to plant roots. Cultivated plants can selectively nurture specific rhizosphere microbial groups through their root secretions, leveraging signaling molecules or unique substrates. This phenomenon is known as the “rhizosphere effect” (Zhalnina et al., 2018; Huang et al., 2019; Rolfe et al., 2019; Zhang G. Z. et al., 2021). This effect articulates how soil microbes are major influencers contributing to the immediate environment of plant roots.

Various aspects including the host genotype, growth cycles, and ecological compartments can markedly impact the composition and functionalities of plant microbial assemblies (Cregger et al., 2018; Gao et al., 2021; Sun et al., 2021; Xiong et al., 2021; Huang et al., 2022). Microbial groups within the soil surrounding plant roots exist across varying ecological compartments such as the rhizoplane, rhizosphere, and bulk soil. Thus, the soil harbors a rich diversity of microbes that are instrumental in maintaining the stability of the ecosystem (Zheng et al., 2019; Ding and Bai, 2021; Li et al., 2021; Huang et al., 2022). Some studies have proposed a multi-step selection model for rhizosphere microbial communities. This suggests that microorganisms are gradually selected across different environments, namely bulk soil, rhizosphere soil, and root, ultimately forming a rhizosphere-specific microbial community (Tian et al., 2020; Zhang et al., 2024). However, most existing studies have considered microbial communities in the root soil as a single entity (Ren et al., 2020; Song et al., 2020; Kui et al., 2021; Shi Y. H. et al., 2022; Wei et al., 2022; Zhang G. Z. et al., 2022). Microbial assembly processes within varying ecological compartments of plant root soils remain unclear. Comprehending the trends of microbial group variations over time in the different compartments is necessary for decrypting the assembly mechanisms of microbiomes in plant root soils. Furthermore, these mechanisms must be elucidated for formulating microbiome-based strategies aimed at augmenting the efficiency and sustainability of plant cultivation (Carrión et al., 2019; Gao et al., 2020; Zhang G. Z. et al., 2022).

Ginseng is obtained from a plants species *Panax ginseng* C. A. Mey. Recognized as the foremost among bulk medicinal herbs, ginseng is typically harvested after 4–5 years of cultivation (Yang et al., 2017; Shi Z. T. et al., 2022). Land used for ginseng cultivation can be used for ginseng replantation only after >30 years (Yang et al., 2004). According to numerous studies, a sustainable cultivation problem might be linked to shifts in soil microbiota (Gao et al., 2021), increased number of pathogenic microorganisms (Weidenhamer, 1996; Wei et al., 2020), deterioration of soil physicochemical characteristics (Vives-Peris et al., 2018), and negative allelopathic effects of ginseng secretions that affect its own growth (Yang et al., 2000, 2017). The aforementioned ginseng-associated problems of sustainable cultivation and shortage of suitable land are primary impediments to ginseng industry’s growth in China (Shen et al., 2015). Consequently, comprehending the spatiotemporal heterogeneity of rhizosphere microecology in ginseng is imperative. This understanding is the basis for adopting standardized ginseng cultivation practices and promoting sustainable ecological development (Ying, 2013). Despite this, our knowledge remains limited regarding the heterogeneity influencing microbial communities in different ecological compartments of ginseng root soil affected by cultivation duration. Current studies on ginseng have primarily investigated the rhizosphere soil (Xiao, 2015; Xiao et al., 2016; Wang et al., 2021; Zhang J. X. et al., 2022), with no details on how microbial communities in the different ecological compartments (namely rhizoplane, rhizosphere, and bulk soil) evolve over varying ginseng cultivation periods. This knowledge gap impedes our understanding of plant–microbe coevolution and the potential to control and use these microbes for sustainable agriculture (Berendsen et al., 2012; Dini-Andreote and Raaijmakers, 2018; Zhang G. Z. et al., 2021).

Lightly disturbed soils have not undergone artificial tillage, have not experienced anthropogenic fertilization interventions, and have not been used to cultivate other crops, which avoids issues such as imbalances in their microbial structure. In this experiment, mildly disturbed soil appropriate for ginseng growth was selected within prime production areas. Here, this soil has been referred to as soil from deforested lands. This soil has deep layers, high organic matter content (7–16%), a loose and breathable structure, strong drainage and water retention abilities, and a high trace element concentration. The predominant types of soil are brown soil and dark brown soil. This soil type is prominently found in mixed coniferous and broad-leaved forests and is formed through years of weathering and accumulation of residual deposits from parent materials such as granite and basalt, mixed with plant residues and fallen leaves (Im et al., 2010; Vendan et al., 2010). Deforested land soil is advantageous for ginseng cultivation because it has a good quality, requires mature cultivation techniques, has fewer pests and diseases, offers higher yields, and allows longer durations of planting. We hypothesized that ginseng in logged forests has a stronger relationship with its root microbial communities because of its long survival time. However, the changing patterns of microbial communities in different root ecological compartments under mildly disturbed soils remain uninvestigated. The simplified backdrop of mildly disturbed soils, excluding influencing factors such as soil compacting, soil-borne diseases, and preceding crops (Shen et al., 2015), allows for the maximal retention of ginseng’s natural state and can be aptly used to study the influence of cultivating ginseng under natural conditions on the heterogeneity of rhizosphere microbial communities. We here investigated the patterns of microbial taxa variations in different ecological compartments over varying cultivation durations. We also mined for microecological factors contributing to the longer survival of ginseng under mildly disturbed soils and explored the correlation between soil physicochemical properties and microbial communities. This study establishes a foundation clarifying how cultivated ginseng affects the heterogeneity of root–soil microbiomes and improvement of ginseng growth years. Deciphering the sustainable cultivation problem of ginseng is crucial. This study seeks to offer a theoretical groundwork for sustainably developing ginseng cultivation.

In this study, we used 4-year (F4) and 5-year (F5) ginseng cultivated in mildly disturbed soils to scrutinize the diversity of bacterial and fungal communities in different ecological compartments, including the rhizoplane (B), rhizosphere (J), and bulk soil (T). Our hypotheses were as follows: (1) Longer ginseng survival in mildly disturbed soils is related to certain microecological factors in its root system. (2) The number of cultivation years significantly influences the microbial diversity and structural composition in the different ecological compartments of the ginseng root, and various signature microorganisms are substantially enriched in the different ecological compartments; and (3) Diverse soil physicochemical properties and enzyme activities, are somewhat correlated to microbial communities in both the rhizosphere and bulk soil.

## 2 Materials and methods

### 2.1 Field experiments and sample collection

All samples were gathered from Yanbian (coordinates: N43°26′51″, E129°32′23″, altitude: 489 m), the key ginseng production region in Jilin Province, Northeast China. The site was formerly a forested area and has now been converted for ginseng cultivation. The region has a temperate continental monsoon climate, with an average annual temperature of 4.9°C, yearly precipitation of 574.9 mm, and annual sunlight of 2,234 h. On September 17, 2021, samples were collected from 4-year-old (F4) and 5-year-old (F5) ginseng plants. The F4 group comprised plants that had been transplanted as 3-year-old seedlings and grown for an additional year after transplantation, while the F5 group consisted of plants that had been transplanted at the same initial age but grown for two additional years after transplantation. Six replicates were maintained for each group, with each replicate containing over 20 healthy ginseng plants, which totaled up to more than 250 ginseng plants.

### 2.2 Sample handling

All experimental utensils were sterilized. After the topsoil layer was removed, intact ginseng plants were carefully excavated and gently shaken to harvest the root system. The soil dislodged during this process was classified as bulk soil (T). The soil samples were sieved through a 20-mesh screen, labeled, promptly placed in sampling boxes fitted with ice packs, and transported to the laboratory for further analysis.

While working on an ultra-clean workbench, we eliminated the soil located within a 2-mm radius surrounding the ginseng root by using a brush. This soil sample was designated as rhizosphere soil (J). Subsequently, each specimen was subjected to ultrasonic shock at 50–60 Hz for (30 s and washed using a PBS buffer solution). The resulting rinsate was subjected to high-speed centrifugation at 6,000 × *g*, 4°C for 20 min to obtain the soil associated directly with the root surface, which was termed rhizoplane soil (B) (Edwards et al., 2015; Qin et al., 2018).

Samples from both years, encompassing six rhizosphere soil replicates and five replicates each of rhizoplane and bulk soils, were bifurcated, yielding a total of 64 sample groups. One sample (F4B, F4J, F4T, F5B, F5J, and F5T) segment was earmarked for 16S rDNA and ITS assays and stored at −80°C for DNA extraction. The remaining sample groups were used for measuring physicochemical attributes and enzyme activities.

### 2.3 Soil physical and chemical properties and enzyme activity tests

We evaluated various soil physicochemical properties including soil water content (SWC), pH, electrical conductivity (EC), organic matter content (OM), total nitrogen (TN), total phosphorus (TP), total potassium (TK), alkaline hydrolyzable nitrogen (AN), and olsen phosphorus (OP). The drying method was used for assessing SWC, potentiometric method for pH, electrode method for EC, direct heating method for OM, Kjeldahl method for TN, sodium hydroxide alkali soluble molybdenum antimony colorimetric method for TP, sodium hydroxide melting method for TK, alkali diffusion method for AN, and ultraviolet–visible spectrophotometry for OP (Bao, 2000).

We quantified the activities of cellulase (S-CL), β-glucosidase (S-β-GC), acid protease (S-AcPr), urease (S-UE), acid phosphatase (S-ACP), sucrase (S-SC), catalase (S-CAT), and dehydrogenase (S-DHA) in the soil samples. All analyses were performed using reagent kits provided by Solarbio (Beijing) Co., thereby ensuring standardized procedures were followed for all measurements.

### 2.4 DNA extraction, PCR amplification, and high-throughput sequencing

The CTAB method was followed for extracting DNA from each sample species, while nuclear-free water was used as a blank control. Total extracted DNA was eluted with 50 μL elution buffer and stored at −80°C until further processing for PCR amplification.

To amplify the V3–V4 region of 16S rDNA, we used the primer set recommended by Logue et al. (2016): 341F (5′-CCTACGGGNGGCWGCAG-3′) and 805R (5′-GACTACHVGGGTATCTAATCC-3′). For ITS, the primers described by Yao et al. (2019) were used: ITS1FI2 (5′-GTGARTCATCGAATCTTTG-3′) and ITS2 (5′-TCCTCCGCTTATTGATATGC-3′). PCR amplification was performed in a 25-μL reaction mixture containing 25 ng template DNA, 12.5 μL PCR Premix, 2.5 μL each primer, and a precise volume of PCR-grade water. The PCR conditions for the amplification of prokaryotic 16S fragments included an initial denaturation at 98°C for 30 s, followed by denaturation at 98°C for 10 s, annealing at 54°C for 30 s, extension at 72°C for 45 s for 32 cycles, and a final extension at 72°C for 10 min. The PCR products were verified through 2% agarose gel electrophoresis. Additionally, instead of the sample solution, ultrapure water was used as a negative control to ensure the PCR results were accurate. The PCR products were purified using AMPure XT beads (Beckman Coulter Genomics, Danvers, MA, USA) and quantified using Qubit (Invitrogen, USA). Finally, all samples were sequenced on the Illumina NovaSeq platform. The sequencing service was provided by LC-Bio, Hangzhou, China.

### 2.5 Data analysis

The samples were sequenced, with corresponding paired-end reads assigned to these samples based on unique barcodes. Later, the barcodes and primer sequences were removed. The paired-end reads were merged using FLASH. Raw data were filtered with fqtrim (v0.94) to ensure high-quality reads. Chimeric sequences were filtered out using Vsearch software (v2.3.4). Subsequently, the relative abundance of bacteria and fungi for each sample was normalized against the feature abundance by using the SILVA (release 138) classifier, RDP database, and UNITE database.

Using the ASV (amplified sequence variant) feature sequence and abundance tables, both alpha and beta diversity analyses were conducted. Microbial abundance and diversity were assessed using Chao1 and Shannon diversity indices. Between-group differences were analyzed using the Kruskal–Wallis rank sum test. Meanwhile, a two-dimensional presentation of difference analysis was performed by conducting principal coordinates analysis (PCoA), based on the Bray–Curtis distance matrix for ASV communities, and combined with multivariate PERMANOVA (also known as Adonis) to analyze the causes of differences in the samples. The permutation test was conducted to test for significance, and replicates with less than two groups of large differences were excluded. Stacked plots were employed to categorize the relative abundance of the TOP30 species, which presented the relative abundance of each group in a different form. The LEfSe analysis of variance was performed to identify biomarkers in each ecological compartment. Then, the Spearman correlation coefficient was used to analyze the relationship between soil chemical properties, enzyme activities, and relative abundance of TOP30 bacteria and fungi corresponding to different cropping years in inter-root soil and soil body and presented as clustered heatmaps. The graphs were primarily generated using “Image GP,” “Easy Amplicon,” “plotrix,” “ggplot2 (3.2.0),” “vegan,” “nsegata-lefse,” and “corrplot” packages in R-3.4.4, while the other graphs were generated using the R package (v3.5.2) (Liu et al., 2023).

The study data were deposited in NCBI under the registration numbers PRJNA1004743 and PRJNA1004746 for bacterial and fungal data, respectively.

## 3 Results

### 3.1 Analysis of composition and changes in the diversity of soil microorganisms

We obtained 2,399,495 bacterial and 2,366,443 fungal valid sequences from 64 samples, which were then categorized into 20,314 bacterial and 6,241 fungal ASVs, respectively.

**Figure 1:**
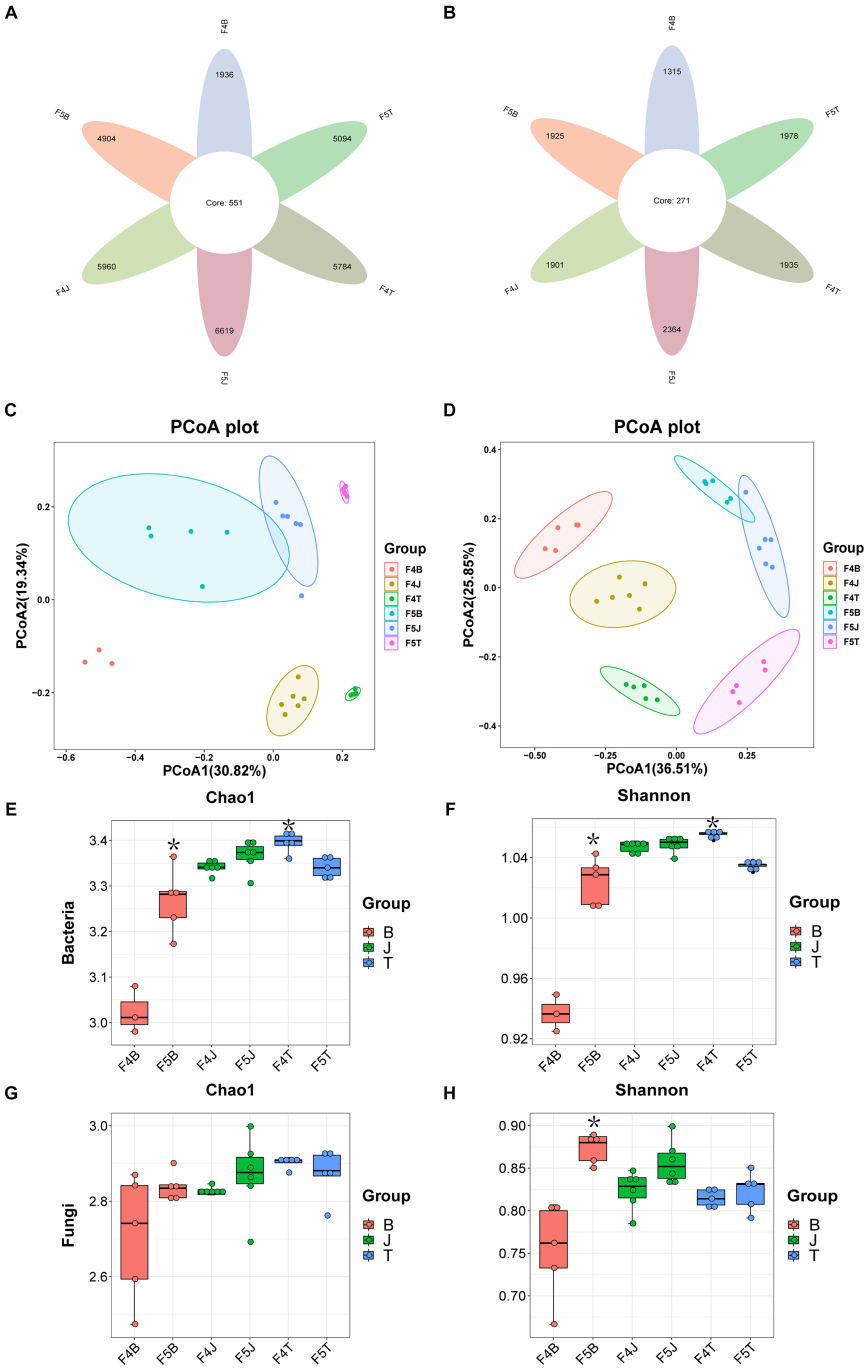
Alpha-diversity and beta-diversity. The petal diagram **(A)** shows bacteria and **(B)** shows fungi. PCoA plots illustrate the community structure β-diversities of bacteria **(C)** and fungi **(D)** across different cultivation years and ecological compartments of ginseng. Chao 1 for bacteria **(E)**, Shannon for bacteria **(F)**, Chao 1 for fungi **(G)**, and Shannon for fungi **(H)**. Data are presented as the mean ± standard error (*p* < 0.05, according to Kruskal–Wallis test).

The number of bacterial ASVs in each soil ecological compartment, except for bulk soil, increased with age. The number decreased by 11.93% for the bulk soil, whereas increased by 153 and 11.06%, respectively, for rhizoplane and rhizosphere soils (Figure 1A). The number of fungal ASVs increased with an increase in the number of planting years, with the highest increase noted in the rhizoplane soil (46.39%), followed by the rhizosphere and bulk soils (24.36 and 2.2%, respectively) (Figure 1B). The number of ASVs of microbial taxa in all ecological compartments generally increased as the planting years increased, except for the decrease in bacteria ASVs noted in the bulk soil. The greatest increase was noted in the rhizoplane soil. The β-diversity results of PCoA unveiled that microbial taxa varied significantly across planting years and ecological compartments and were significantly separated on PCoA2 and PCoA1 axes, respectively (Figures 1C,D), with 50.16% explained by bacteria and 62.36% by fungi.

According to the Chao1 index of species richness and Shannon index of diversity for α-diversity, the bacterial diversity of the rhizoplane soil (Chao1 and Shannon indices) was the lowest among all ecological compartments (Figures 1E,F). The bacterial diversity (Chao1 and Shannon indices) and fungal diversity (Shannon index) of the rhizoplane soil increased significantly and substantially with each planting year (Figures 1E,F,H). By contrast, the bacterial diversity (Chao1 and Shannon indices) of the bulk soil decreased significantly with each planting year (Figures 1E,F). Conversely, bacterial and fungal diversities (Chao1 and Shannon indices) of the rhizosphere soil was relatively stable without significant differences across planting years (Figures 1E–H).

**Figure 2:**
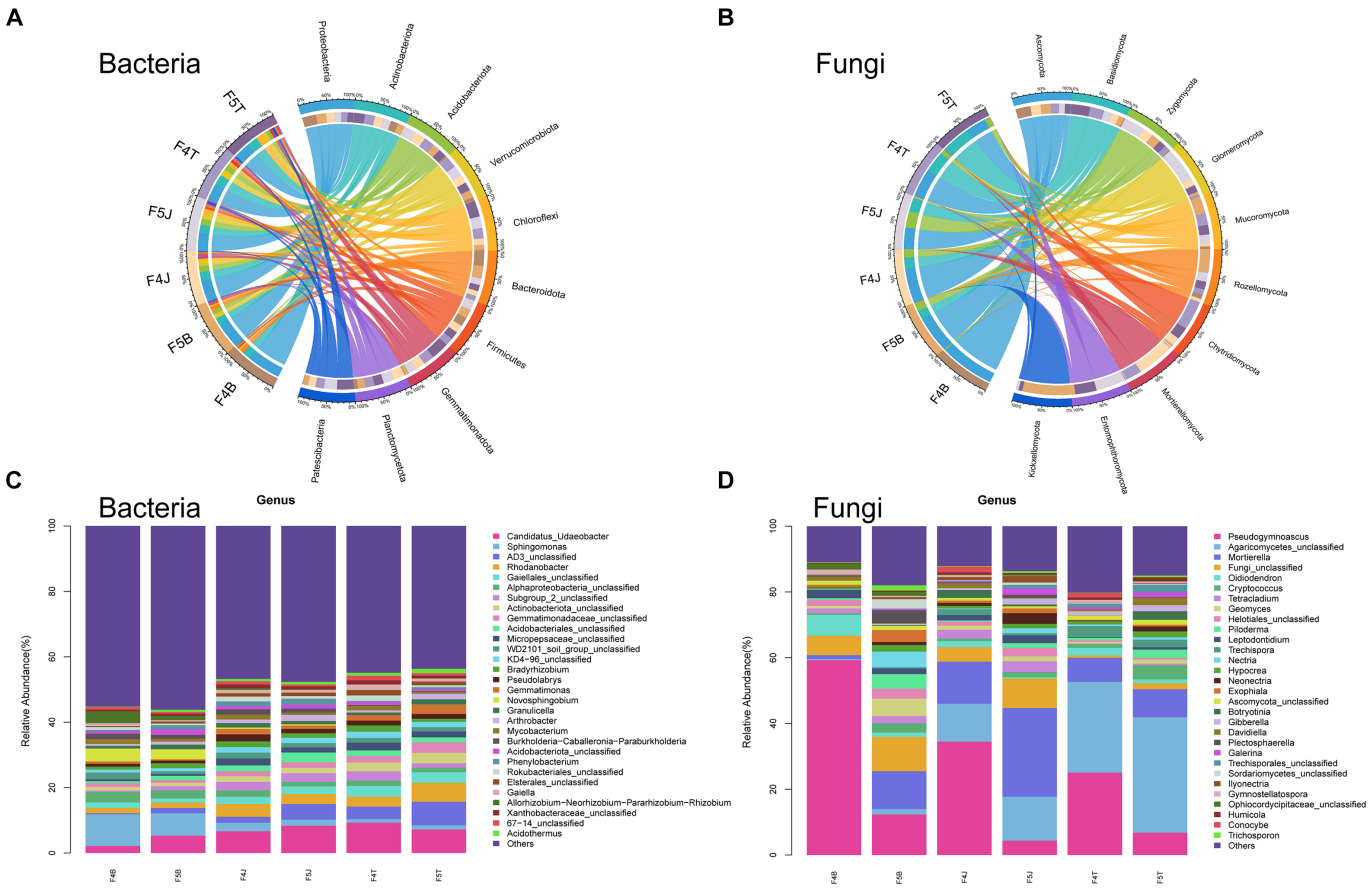
Species abundance chart. Circos comparing the abundances of the top 10 bacterial phyla **(A)** and fungal phyla **(B)**. Stacked column charts comparing the top 30 bacterial genera **(C)** and fungal genera **(D)**.

The PERMANOVA analysis unveiled that F4 had a greater explanatory power for microbiome composition differences across various ecological compartments, with a larger impact noted on bacteria than on fungi (bacteria R^2^ = 0.6940, fungi R^2^ = 0.6543, *p* = 0.001, Table 1). As the cultivation years increased, the explanatory power diminished, with fungi being more affected than bacteria (bacteria R^2^ = 0.5126, fungi R^2^ = 0.6235, *p* = 0.001, Table 1). Moreover, the cultivation duration had the least effect on the rhizosphere soil microbiome (bacteria R^2^ = 0.3742, fungi R^2^ = 0.5930, *p* = 0.003, Table 1), whereas the greatest effect was noted on the rhizoplane soil fungi (R^2^ = 0.6597, *p* = 0.01, Table 1) and bulk soil bacteria (bacteria R^2^ = 0.6623, *p* = 0.005, Table 1).

**Table 1:** PERMANOVA analysis based on the Bray–Curtis distance.

Sample grouping	Ecological niches F4 (B vs. J vs. T)		Ecological niches F5 (B vs. J vs. T)		Rhizoplane B (F4 vs. F5)		Rhizosphere J (F4 vs. F5)		Bulk Soil T (F4 vs. F5)	
Df	2		2		1		1		1	
Microbiota	Bacteria	Fungi	Bacteria	Fungi	Bacteria	Fungi	Bacteria	Fungi	Bacteria	Fungi
R^2^	0.6940	0.6543	0.5126	0.6235	0.4940	0.6597	0.3742	0.5930	0.6623	0.6162
Pr(>F)	0.001	0.001	0.001	0.001	0.02	0.01	0.003	0.003	0.005	0.009

R^2^ indicates the proportion of variation explained by the different groupings, and *p* < 0.05 indicates a high confidence level in the generalizability of the test results.

### 3.2 Shifts in soil microbial community composition across ecological compartments over cultivation time

At the phylum level, the dominant bacterial phyla with a relative abundance of >1% included 9 types of bacteria, namely Proteobacteria (29.43–48.80%), Actinobacteria (13.58–20.31%), Acidobacteria (7.97–12.74%), Verrucomicrobiota (4.54–12.11%), Chloroflexi (2.58–11.35%), Bacteroidota (2.66–6.86%), Gemmatimonadota (1.25–6.04%), Planctomycetota (2.16–4.32%), and Firmicutes (2.42–3.14%). Among them, the relative abundance of Proteobacteria decreased in all ecological compartments in F5 compared with F4, whereas those of Chloroflexi, Bacteroidota, and Firmicutes increased in all ecological compartments in F5. The relative abundances of Actinobacteria and Verrucomicrobiota increased in the rhizoplane and rhizosphere soils with an increase in planting years and decreased in the bulk soil over the same period. Conversely, Acidobacteria, Gemmatimonadota, and Planctomycetota exhibited an opposite trend to that of Actinobacteria, with their relative abundances decreasing in the rhizoplane and rhizosphere soils and increasing in the bulk soil with an increment in planting years (Figure 2A).

**Figure 3:**
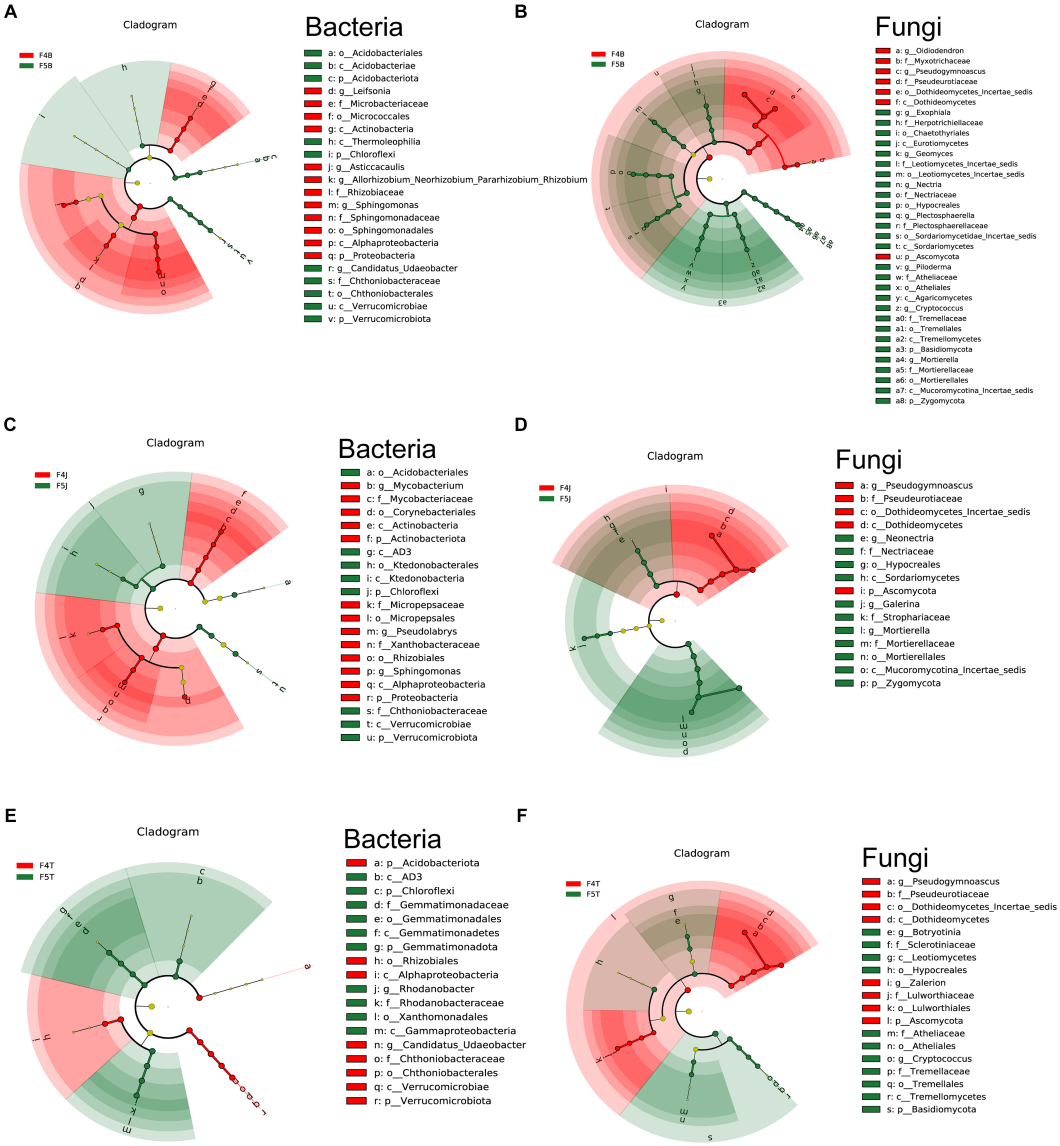
LEfSe analysis of different planting years of ecological compartments in ginseng root soil samples. Arcograms depicting taxonomic evolutionary branches indicate two groups: bacteria **(A,C,E)** and fungi **(B,D,F)**. Each arcogram comprises concentric circles representing six taxonomic levels: Phylum, Class, Order, Family, Genus, and Species, from the innermost to outermost. Within each arcogram, nodes represent species classifications at their respective taxonomic levels. Node colors indicate their significance in comparison groups. Yellow nodes denote species with no significant difference in the comparison group, red nodes denote species that act as biomarkers in 4-year-old ginseng samples, and green nodes indicate species that act as biomarkers in 5-year-old ginseng samples. The gates of significant differences are labeled directly on the figure, and distinctions in species nodes at other levels are identified by letters (All LDA > 4.0, except F4J vs. F5J bacteria LEfSe LDA > 3.5).

The dominant fungal phyla with a relative abundance of >1% were Ascomycetes (36.78–89.31%), Basidiomycota (3.14–52.58%), and Zygomycota (1.4–27.57%). Ascomycota was dominant in all ecological compartments, with a decrease in its relative abundance in all compartments with an increase in the planting years. By contrast, the relative abundances of Zygomycota and Basidiomycota increased in all compartments with more extended planting durations. Particularly, the relative abundance of Basidiomycota substantially increase in F5B, and that of Zygomycota significantly increased in both F5B and F5J as the planting years increased (Figure 2B).

At the genus level, the dominant bacteria with a relative abundance of >1% were *Candidatus Udaeobacter* (2.17–9.33%), *Sphingomonas* (1.03–9.67%), and *Rhodanobacter* (1.61–5.75%). Among them, the relative abundance of *Candidatus Udaeobacter* increased in the rhizoplane and rhizosphere soils as the planting years progressed, but decreased in the bulk soil over the same period. Conversely, the relative abundance of *Sphingomonas* declined in the rhizoplane and rhizosphere soils, whereas increased in the bulk soil with an increase in the planting years. Except in the rhizosphere soil, the relative abundance of *Rhodanobacter* increased in the rhizoplane and bulk soils as the planting years extended. Moreover, the relative abundance of *Novosphingobium* was higher in the rhizoplane soil, albeit decreasing as the planting years increased (Figure 2C).

The dominant fungal genera, with a relative abundance of >1%, included *Pseudogymnoascus* (4.44–67.10%) and *Mortierella* (1.07–26.99%). *Pseudogymnoascus* was the dominant fungal genus in all ecological compartments of F4 (B: 59.26%, J: 34.51%, T: 25.11%), but its relative abundance substantially reduced as the cultivation period lengthened (B: 12.36%, J: 4.44%, T: 6.80%). Concurrently, the relative abundance of the potentially beneficial *Mortierella* fungus increased in all ecological compartments, notably increasing remarkably in the rhizoplane and rhizosphere soils (F4B: 1.23%, F5B: 11.53%, F4J: 12.79%, F5J: 26.98%, F4T: 7.44%, F5T: 8.53%). The relative abundances of other fungi, such as *Oidiodendron* and *Trechispora*, decreased across all ecological compartments in F5, with the relative abundance of the potentially beneficial fungus *Oidiodendron* significantly decreasing in F5B.

By contrast, the relative abundances of numerous potentially pathogenic fungi including *Cryptococcus* (F4B: 0.52%, F5B: 2.83%), *Neonectria* (F4B: 0.12%, F5B: 0.89%), *llyonectria* (F4B: 0.09%, F5B: 0.56%), *Gibberella* (F4B: 0.06%, F5B: 0.41%), *Piloderma* (F4B: 0.47%, F5B: 4.44%), and *Plectosphaerella* (F4B: 0.44%, F5B: 3.88%) increased across all ecological compartments as the planting years progressed. In particular, the relative abundance in the F5B soil increased by 4.44–8.44 times (Figure 2D).

### 3.3 Differences in soil microbial communities in different cultivation years

In the rhizoplane soil, F4B notably enriched 14 bacterial types, including potentially beneficial Proteobacteria (genera: *Sphingomonas, Asticcacaulis*, and *Allorhizobium-Neorhizobium-Pararhizobium-Rhizobium* complex) and Actinobacteriota (genus: *Leifsonia*). F5B enhanced 10 species from the phyla Chloroflexi, Verrucomicrobiota (genus: *Candidatus Udaeobacter*), and Actinobacteriota (class: Thermoleophilia, order: Acidobacteriales) (Figure 3A). F4B harbored 9 fungi including potentially beneficial fungi from the Ascomycota phylum (genera: *Oidiodendron* and *Pseudogymnoascus*), and F5B harbored 32 fungal taxa from the Ascomycota, Basidiomycota, and Zygomycota phyla (Figure 3B).

In the rhizosphere soil, F4J significantly enriched 13 bacterial types from Proteobacteria (genera: *Pseudolabrys* and *Sphingomonas*, and family: Micropepsaceae) and Actinobacteriota (genus: *Mycobacterium*). F5J hosted 8 bacteria from Chloroflexi (class: AD3, order: Ktedonobacterales), Verrucomicrobiota (family: Chthoniobacteraceae), and Acidobacteriota (order: Acidobacteriales) (Figure 3C). F4J enriched 7 species mainly from the Ascomycota phylum, and F5J hosted 14 fungal taxa including potentially harmful fungi from Ascomycota (genus: *Neonectria*) and others from Zygomycota and Basidiomycota (Figure 3D).

In the bulk soil, F4T significantly enhanced 8 bacterial types from Proteobacteria (order: Rhizobiales), Verrucomicrobiota (genus: *Candidatus Udaeobacter*), and Acidobacteriota. F5T fostered 10species from Chloroflexi (class: AD3), Proteobacteria (genus: *Rhodanobacter*), and Gemmatimonadota (family: Gemmatimonadaceae) (Figure 3E). F4T enriched 11 fungi, largely from Ascomycota, and F5T hosted 11 species from Ascomycota and Basidiomycota, featuring genera such as *Botryotinia* and *Cryptococcus* (Figure 3F). Supplementary Figures S1, S2 present the microbiome differences among the different ecological compartments.

### 3.4 Correlation cluster marker heatmap analysis

To delve deeper into the relationships between soil microorganisms, soil physicochemical properties, and enzyme activities, we selected the top 30 bacterial and fungal genera for the correlation cluster marker heatmap analysis.

**Figure 4:**
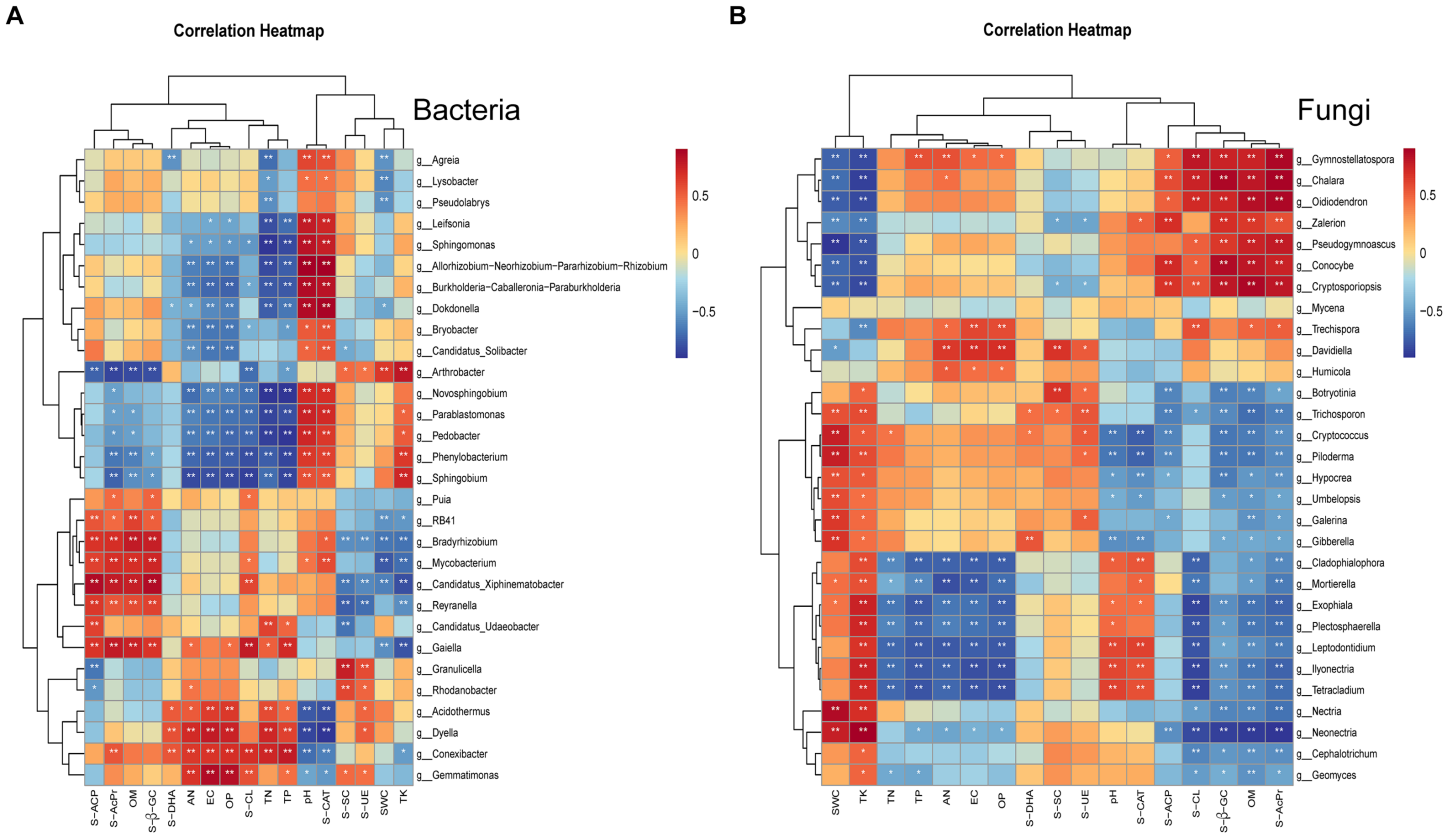
Clustered heatmap of correlations between the top 30 microbial groups and environmental indicators. In the heatmap, columns represent species, and rows represent environmental indicators. The Spearman correlation coefficients calculated between bacteria **(A)** and fungi **(B)** and the indicators are depicted, with “*” denoting *p* < 0.05 and “**” denoting *p* < 0.01. Red indicates a positive correlation, whereas blue indicates a negative correlation.

The bacteria were chiefly influenced by pH and S-CAT levels. pH particularly had a considerable impact on numerous potentially beneficial bacteria, including those belonging to genera such as *Sphingomonas*, *Novosphingobium*, and *Spingobium*. Thus, bacterial dynamics can be modulated by controlling pH and monitoring S-CAT (Figure 4A).

By contrast, fungi were predominantly affected by SWC and TK levels. Notably, SWC was significantly correlated with potentially beneficial fungal genera such as *Mortierella* and the ectomycorrhizal *Piloderma*, as well as with detrimental fungi such as *Gibberella* and *Exophiala*. Furthermore, pH had notable associations with potentially harmful fungi such as *Ilyonectria*, *Exophiala*, and *Leptodontidium*. Similarly, TK was significantly correlated with both potentially beneficial fungi (*Mortierella*, *Exophiala*) and potentially harmful fungi (*Piloderma*, *Ilyonectria*, *Gibberella*, and *Leptodontidium*) (Figure 4B). These findings highlight potential pathways for managing fungal populations in cultivated ginseng soil by manipulating factors such as pH, SWC, and TK levels.

## 4 Discussion

### 4.1 Planting year and ecological compartment significantly influence microbial community diversity of ginseng root soil

Several factors can affect the microbial community diversity, including the host genotype (Wang et al., 2021), soil conditions (Jiang et al., 2021), ecological compartment (Gao et al., 2021), planting duration (Laforest-Lapointe et al., 2016), and growth phase (Huang et al., 2022). We here sourced all samples from a single land plot that was previously a forested area and is growing a common ginseng variety. As the samples were collected in the fall during the root expansion stage, the influences of different varieties, soil environments, and growth stages on the microbial communities were eliminated. On investigating bacterial and fungal communities in the rhizosphere soil of 4 ginseng cultivars, namely Gaoli, common, Shizhu, and Biantiao ginsengs, Wang et al. (2021) found that the cultivars affected the composition and diversity of rhizosphere soil microbial communities. Based on this finding, we further underscored that both the planting duration and ecological compartments significantly affect microbial communities in the soil surrounding ginseng roots (Figure 1).

A previous study conducted by our team revealed that the soil microbial diversity in ginseng transplant areas gradually reduced as the planting duration increased (Xiao, 2015; Xiao et al., 2016). Our present study goes a step further to unveil that different ecological compartments in the soil surrounding ginseng roots have markedly different microbial community diversities, which depends on the planting duration. With an increase in the planting duration, the bacterial diversity and ASV number in the soil hosting the cultivated ginseng decreased. Conversely, the microbial diversity and ASV number in the rhizoplane soil increased. Importantly, compared with those in the rhizoplane and bulk soils, the microbial communities in the ginseng rhizosphere had the greatest stability, exhibiting limited sensitivity to the age of the ginseng plant (Table 1). Huang et al. (2019) demonstrated that triterpenoid compounds participate in the coevolution between plants and rhizosphere microbiomes and can selectively regulate rhizosphere microbes. This finding further accentuates the pivotal role and inherent resilience of rhizosphere microbial communities in agricultural ecosystems.

### 4.2 Impact of planting duration on the composition of microbial communities in different ecological compartments of ginseng root soil

Our study illuminates how microbial taxa in various ecological compartments of ginseng root soil vary with the planting duration. Thus, planting duration is a significant determinant in shaping the microbial community composition within root soil compartments. Different ecological compartments can harbor distinct specific taxa within communities. These communities undergo changes as the planting duration extends. This phenomenon aligns with observations made in other plant studies, thus highlighting the significance of various ecological compartments in selectively recruiting specific microbial taxa across different plant life stages (Huang et al., 2022). Ying et al. (2012) and Wang et al. (2021) have identified Proteobacteria and Acidobacteriota as the dominant bacterial groups in the ginseng rhizosphere. Our team’s earlier analyses, which was conducted using DGGE fingerprinting of 16S rDNA amplification products derived from ginseng soil samples, revealed a primary bacterial composition including phyla such as Proteobacteria, Actinobacteriota, Verrucomicrobiota, and Acidobacteriota (Xiao, 2015; Xiao et al., 2016). Our recent studies have illustrated that the ginseng root soil has a rich bacterial diversity encompassing 9 phyla, such as Chloroflexi, Bacteroidota, Firmicutes, and Gemmatimonadota (Figure 2A) Our findings resonate with those of Wang et al. (2021), pinpointing Basidiomycota, Ascomycota, and Zygomycota as the prevalent fungal groups in the ginseng rhizosphere (Figure 2B). Furthermore, phyla, such as Basidiomycota and Ascomycota, enhance resistance against pathogens and promote tolerance to abiotic stresses (Mardanova et al., 2019). Thus, microbial taxa across different ecological compartments vary with the years of cultivation. This emphasizes the compartment-specific interactions during ginseng growth, thereby clarifying a nuanced understanding of the complex microbial dynamics in ginseng cultivation.

### 4.3 Root soil microorganisms associated with ginseng pathogenicity and survival time in mildly disturbed soil

We used soil from a logged forest area for this experiment. This area is renowned for its high organic matter content, substantial water retention capacity, moderate pH, and low prevalence of soil-transmitted diseases. These factors have created an environment potentially favorable for long-term ginseng cultivation (Shen et al., 2015). Our findings revealed that ginseng planted in this soil type for extended periods exhibited characteristics identical to those having a prolonged survival time. This includes a notable enrichment of the *Mortierella* genus. The relative abundance of *Mortierella* significantly increased, particularly in the rhizoplane and rhizosphere soils, with an extension of the planting duration. This increase was parallel to a significant reduction in the relative abundance of the phenolic acid-producing bacterium *Pseudogymnoascus* (Figures 2C,D, 3B,D,F; Guo et al., 2019). The heightened abundance of *Mortierella* has been linked to longer survival times of ginseng (Wang et al., 2021). *Mortierella* is a critical indicator useful for preventing and controlling ginseng fusarium wilt disease, with certain species serving as potential antagonists against various plant pathogens owing to their ability to produce antibiotics (Somers et al., 2004). Furthermore, recent genomic sequencing unveiled that some *Mortierella* species can synthesize and breakdown a diverse array of chemical compounds (Li et al., 2023), thereby indicating that *Mortierella* can resist pathogens and degrade some autotoxic substances secreted by ginseng. This suggests a promising avenue for developing biocontrol fungi to augment ginseng survival time.

Our team’s earlier analyses reported the presence of potentially pathogenic fungi, such as *Cryptococcus* and *Fusarium* fungal genera, exclusively in cultivated ginseng soil (Xiao, 2015; Xiao et al., 2016). Further investigations unveiled that as the cultivation years increase, various fungi associated with root diseases in ginseng and other plants, including *Cryptococcus*, *Plectosphaerella,* and *Piloderma*, are primarily enriched in F5B (Figure 3B). Conversely, potentially beneficial bacteria such as *Sphingomonas* and *Allorhizobium-Neorhizobium-Pararhizobium-Rhizobium* were significantly enriched in F4B. This pattern suggests that an increase in the ginseng cultivation duration results in a decline in the abundance of potentially beneficial microbes, whereas a rise in the abundance of potentially harmful microbes in the rhizoplane soil. This microbial imbalance at the root surface compartment might be a reason for the observed continuous cropping obstacles. These findings clarify why Biantiao ginseng can be cultivated for prolonged periods in the Tonghua region of Jilin. Because ginseng farmers remove the fibrous roots during transplantation, a traditional practice rooted in wisdom accumulated over generations, the attachment of numerous potential harmful microorganisms to the substantial root surface area of the roots is reduced. This significantly enhances the ginseng seedling survival rate and growth cycle.

### 4.4 Effect of physicochemical properties on the microbial taxa of ginseng roots in mildly disturbed soil

Factors such as soil pH and nutrient levels can modulate the aggregation of rhizosphere bacterial and fungal communities to fulfill the plant-required functionalities (Rousk et al., 2010; Ren et al., 2020; Zhang Y. et al., 2021). By leveraging this assembly principle, microbial populations present in plant root systems can be steered, thereby enhancing agricultural sustainability (Ren et al., 2020). Jiang et al. (2021) reported the significant influence of soil NH4 + -N and pH on microbial community fluctuations (*p* < 0.05). We noted that soil pH is a pivotal determinant influencing bacterial taxa within the ginseng root system (Figure 4A). This finding corroborates with those of other studies (Lauber et al., 2009; Jiang et al., 2021). We noted a heightened sensitivity of bacteria to pH alterations compared with fungi. Additionally, soil pH and S-CAT in cultivated ginseng environments were positively correlated with several potentially beneficial bacterial genera including *Sphingomonas*, *Novosphingobium*, and *Spingobium*. Thus, the bacterial microecological equilibrium during ginseng cultivation can be maintained through vigilant controlling of pH and monitoring of S-CAT metrics. Further insights from Lee and colleagues indicate the ginseng seedling root weight, number of quality seedlings, and moisture content of seedbed soil are significantly positively correlated (Shen et al., 2015). Xian et al. (2022) identified TK as a primary factor influencing fungal composition in the rhizosphere soil of Huachonglou roots. Based on the seminal work of Dennis et al. (2012), who identified total C/N as a major factor affecting the soil fungal community dynamics, with soil pH playing a secondary role, the present study accentuates the impact of SWC and TK on fungal taxa (Figure 4B). Herein, substantial correlations were observed between moisture content and both potentially beneficial (*Mortierella* and *Piloderma*) and potentially detrimental (*Gibberella*) fungi. Moreover, pH was significantly associated with potentially harmful fungi including *Ilyonectria*, and *Leptodontidium*, while TK was substantially correlated with both potentially beneficial (*Mortierella* and *Exophiala*) and potentially harmful (*Piloderma*, *Ilyonectria*, *Gibberella*, and *Leptodontidium*) fungi. This denotes that the soil fungal dynamics of the cultivated ginseng can be modulated by adjusting soil pH, SWC, and TK. However, variations rooted in plant genotypes and environmental factors engender distinct differences in this regard.

## 5 Conclusion

The present study elucidates that the cultivation duration significantly affects the microbial community diversity and composition in various ecological compartments of ginseng root soil systems under lightly disturbed soil conditions. Notably, as the cultivation years increased, ginseng exhibited a substantial enrichment of the potentially beneficial fungal genus *Mortierella* in rhizoplane and rhizosphere soils. This genus augmented ginseng longevity by resisting pathogens and mitigating the effects of ginseng-secreted autotoxic substances, thus presenting promising avenues for developing biocontrol fungi. As planting years increase, the microecological balance of each ecological compartment may be gradually disrupted, predominantly in terms of fungal taxa, with the rhizoplane soil facing the greatest challenge. Two years after transplantation, the abundance of potentially harmful fungal representatives such as *Cryptococcus* increased in the rhizoplane soil. This finding advocates for adopting measures to diminish the attachment of detrimental microbes to ginseng fibrous roots during transplantation, thereby augmenting both the survival and growth duration of ginseng seedlings. Microbial assemblies in the rhizosphere thus played a pivotal role and were inherently resilient. This reaffirmed their central role in agricultural ecosystems. Thus, focusing on the ecological dimensions of rhizoplane and rhizosphere soils is imperative for emphasizing their significant implications in sustaining ginseng cultivation. Moreover, we here delineated the feasibility of efficiently modulating rhizosphere microbial populations by managing/manipulating physicochemical attributes including pH, SWC, and TK levels, thus offering a practical method for nurturing healthier microbial environments in ginseng cultivation settings. In summary, this study furthers the understanding of microbial community structures in mildly disturbed ginseng soils, which are correlated with the cultivation duration. It furnishes the theoretical scaffolding to improve the lifespan of ginseng plants, navigating toward a sustainable trajectory in traditional Chinese medicine cultivation.

## Data availability statement

The datasets presented in this study can be found in online repositories. The names of the repository/repositories and accession number(s) can be found in the article.

## Author contributions

ZS: Writing – original draft, Writing – review & editing. MY: Writing – original draft. KL: Writing – original draft. LiY: Writing – original draft. LimY: Writing – review & editing. MH: Writing – review & editing.
