# Supplementary figures and images for: Temporal heterogeneity of the root microbiome in Panax ginseng soils across ecological compartments under mild soil disturbance
Source: Front Microbiol. 2024 Jun 11;15:1340575. doi: 10.3389/fmicb.2024.1340575 (PMC11196636; doi:10.3389/fmicb.2024.1340575)

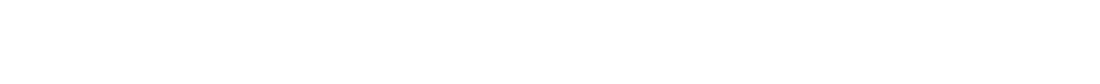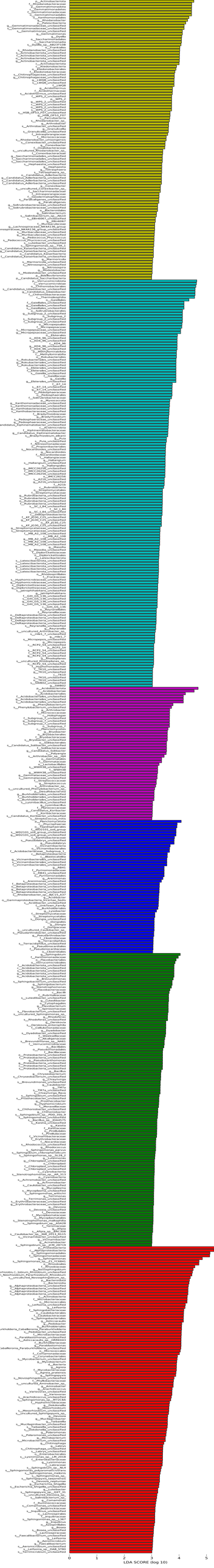

Supplement: Supplementary file 1 [file Data_Sheet_1.PDF]

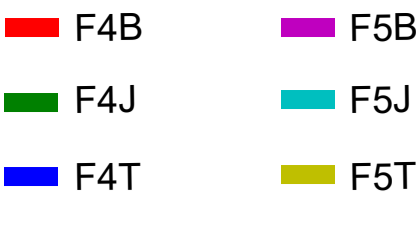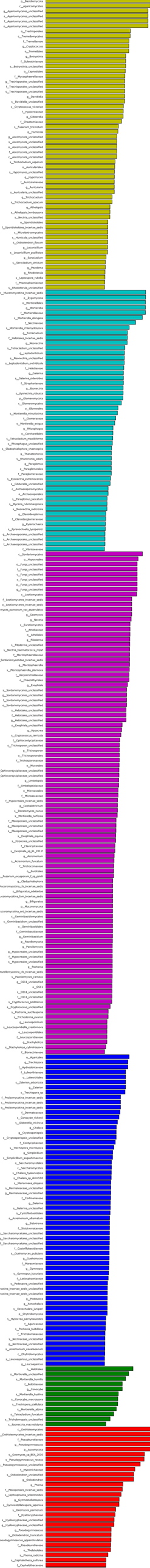

Supplement: Supplementary file 2 [file Data_Sheet_2.PDF]
